# Persistent trigeminal artery variant functioning as a duplicate superior cerebellar artery

**DOI:** 10.1007/s00276-025-03673-1

**Published:** 2025-06-23

**Authors:** George Triantafyllou, Panagiotis Papadopoulos-Manolarakis, George Tsakotos, Anastasia Triantafyllou, Maria Piagkou

**Affiliations:** 1 Department of Anatomy, School of Medicine, Faculty of Health Sciences, National and Kapodistrian University of Athens, 75 Mikras Asias Str., Goudi, 11527 Athens, Greece; 2 Department of Neurosurgery, General Hospital of Nikaia-Piraeus, Athens, Greece

**Keywords:** Persistent trigeminal artery, Superior cerebellar artery, Persistent trigeminal artery variant, Carotid-vertebrobasilar anastomosis, Anatomy, Variation

## Abstract

The cerebral arterial circle displays significant variation, with infrequent configurations often ascertained through contemporary imaging techniques. We present a sporadic case of a duplicated superior cerebellar artery (SCA) discovered during magnetic resonance angiography (MRA) of a 58-year-old female patient. Typical SCA emanates from the distal basilar artery (BA), whereas a duplicate one originates from the pre-cavernous segment of the internal carotid artery (ICA). This accessory vessel traveled parallel to the primary SCA, supplying a portion of its standard vascular territory. This distinctive configuration corresponds to a PTA variant featuring a duplicate SCA, a combination very rarely reported in existing literature. The variant above underscores the embryological origins of the cerebral arterial circle, particularly involving carotid-vertebrobasilar anastomoses. The existence of both a typical and a duplicate SCA constitutes an unprecedented configuration within literature. These vascular anomalies possess clinical significance, especially to neurovascular compression syndromes, such as trigeminal neuralgia, or during surgical and endovascular interventions.

## Introduction

Various arterial variants of the human cerebral arterial circle, including standard and uncommon types, are described during computed tomography angiography (CTA), magnetic resonance angiography (MRA), or digital subtraction angiography (DSA).

Among the distinct variants of the cerebral circulation are the persistent embryonic vessels, including the anastomoses of the carotid-vertebrobasilar system. The most prevalent variant is the persistent trigeminal artery (PTA), which exhibits a pooled prevalence of 0.4%, indicating its rarity as a variation [2]. Nonetheless, this distinctive artery presents several variants, including its fusion with the cerebellar arteries [10].

The cerebellum receives blood supply from three bilateral arteries: the superior cerebellar artery (SCA), which arises from the distal basilar artery (BA); the anterior inferior cerebellar artery (AICA), originating from the proximal BA; and the posterior inferior cerebellar artery (PICA), originating from the distal vertebral artery (VA). The morphological variability of these vessels is frequently noted.

The current anatomical-imaging report describes a rare case of a unilateral duplicate SCA with variant origin.

## Anatomic variation

During an angiographic study based on archived MRA, the scan of a 58-year-old female patient was investigated. The data was obtained from the General Hospital of Nikaia-Piraeus after receiving ethical approval from the relevant authorities (protocol number: 56485, date of approval: 13.11.2024). The scans were documented using Horos software version 3.3.6 (Horos Project). Similar to previous studies, evidence was gathered from the multiplanar reconstruction of the axial, coronal, and sagittal slices and their three-dimensional volume reconstruction.

**Fig. 1:**
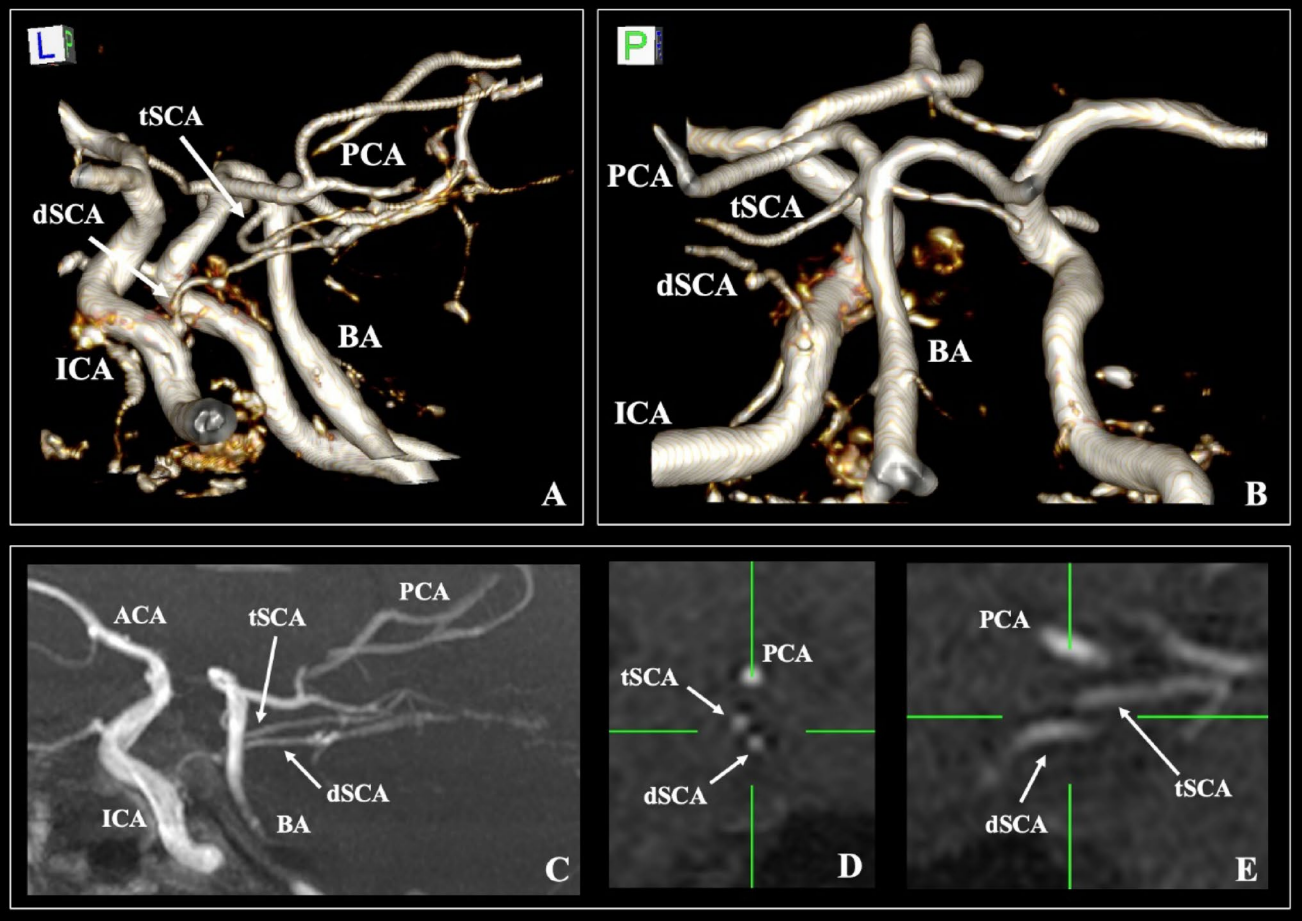
The cerebral arterial circle of the patient. Three-dimensional reconstruction (**A**, **B**), sagittal slice with maximum intensity projection (C), coronal and sagittal slices (**D**, and **E**). The duplicate superior cerebellar artery (dSCA) originates from the internal carotid artery (ICA), and the typical SCA (tSCA) arises from the basilar artery (BA). PCA—posterior cerebral artery, and ACA—anterior cerebral artery

During the three-dimensional reconstruction of the cerebral arterial circle, an accessory artery was observed arising from the pre-cavernous segment of the internal carotid artery (ICA) (diameter of 1.91 mm). The vessel followed a trajectory similar to the typical SCA, located 1.4 mm inferior. The sagittal slices revealed that the accessory vessel supplied part of the SCA territory; thus, this variant corresponded to a duplicate SCA, with one vessel arising from the distal BA and the duplicate one from the ICA (Fig. 1). Further discourse is undertaken regarding the terminology associated with the accessory vessel. The rest of the cerebral arterial circle was free of variants.

## Discussion

**Fig. 2:**
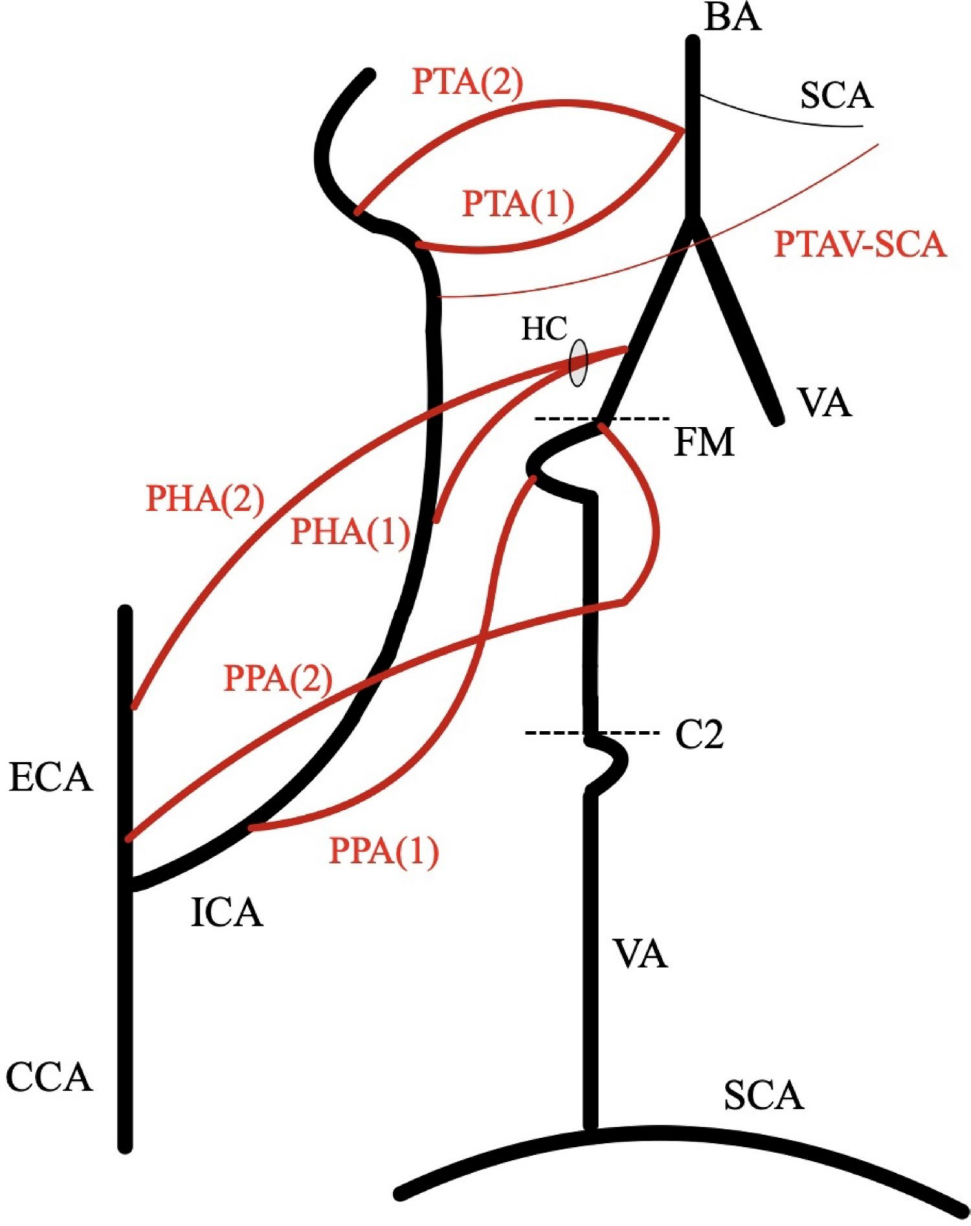
The anatomical possibilities of persistent embryonic anastomoses between the internal carotid artery (ICA) and the vertebrobasilar systems, adapted from [10]. The typical arteries and levels are depicted with black color, the persistent vessels are depicted with red color. PPA(1)—persistent proatlantal artery from the ICA, PPA(2)—persistent proatlantal artery from the external carotid artery (ECA), PHA(1)—persistent hypoglossal artery from the ICA, PHA(2)—persistent hypoglossal artery from the ECA, PTA (1)- persistent trigeminal artery lateral type, PTA(2)—persistent trigeminal artery medial type, PTAV-SCA, persistent trigeminal artery variant- superior cerebellar artery type. VA—vertebral artery, BA—basilar artery, CCA—common carotid artery, SCA- superior cerebellar artery, C2—second cervical vertebra level, FM—foramen magnum level

The most comprehensive manuscript on the embryological development of the cerebral arterial circle was illustrated by Padget [6]. The author delineated four categories of carotid-vertebrobasilar anastomoses that manifest during the fifth week of gestation (with an embryonic length ranging from 5 to 6 mm). Arranged from caudal to cranial, these vessels comprise the proatlantal intersegmental artery, hypoglossal artery, otic artery, and trigeminal artery (Fig. 2). Generally, these arteries undergo regression or fusion with adjacent vessels, establishing the mature cerebral arterial circle [6]. Contemporary imaging techniques facilitate the visualization of these primitive arteries when they persist in adulthood [10]. The typical lateral-type PTA refers to a vessel that connects the BA (most commonly between the AICA and SCA origins) with the ICA (most widely the pre-cavernous segment) [2]. This typical configuration can be found with an estimated pooled prevalence of 0.3% [2]. Variants of the PTA can also be found with a calculated pooled prevalence of 0.2% [2]. These variants correspond to the anastomosis of the PTA with one of the cerebellar arteries. The AICA, which originates from the ICA pre-cavernous segment, is recognized as the most prevalent PTA variant, followed in frequency by the PICA and the SCA. In instances where this variation occurs, the respective cerebellar artery arises directly from the ICA, devoid of any connection to the BA [2].

In this instance, an uncommon anatomical configuration was presented. The SCA was recognized as arising from the BA, which is characteristic; concurrently, a redundant SCA was noted, originating from the pre-cavernous segment of the ICA, indicative of a PTA variant. Notably, in the existing literature, when the SCA is observed to arise from the ICA, the typical SCA is not present from the BA. Consequently, this situation pertains to a duplicate SCA, one variant of which corresponds to the PTA variant. Previously, only Uchino [11] described a similar case observed during MRA involving a 74-year-old female patient. Consequently, this represents another documented case in the literature illustrating this exceptionally rare variation.

This variation was previously recorded for the other cerebellar arteries in an analog modality. Recently, Endo et al. [4] described a unique PTA variant as a duplication of the AICA during DSA of a 51-year-old female patient. Moreover, Lee et al. [5] recorded another PTA variant as a duplicate of the PICA during the DSA of a 51-year-old female patient. Therefore, it is also uncommon to identify other cerebellar arteries with PTA variation and duplication.

The PTA and SCA variants have important clinical implications because of their close spatial relationship with other neurovascular structures. Identifying PTA and its variants is essential before undertaking neurosurgical or endovascular interventions. The typical lateral-type PTA is located near the trigeminal, oculomotor, trochlear, and abducens nerves; as a result, it has been linked to trigeminal neuralgia and ocular motor nerve palsies [2]. Trigeminal neuralgia was also associated with the variants of PTA and SCA [2]. In particular, the SCA duplication was identified as a risk factor for nerve compression, as the accessory vessel could cause neuralgia due to its abnormal course [1, 3]. Due to the high clinical significance, Rusu et al. [7] presented in great detail, during cadaveric dissections, the anatomical relationships of the vascular structures with the trigeminal nerve at the cerebellopontine angle. Other studies referred to the topography of the SCA with the trochlear nerve [8] and the oculomotor nerve [9]. All these anatomical studies concluded to the variable and close relationship of the SCA and PTA with the nerves that could ultimately lead to compression syndromes [7–9]. Nevertheless, SCA aberrant origins, such as the PCA [1] or the ICA (current case), could explain unusual strokes with cerebellar infractions.

In conclusion, identifying a PTA variation as a duplicate SCA was documented during an MRA scan of a 58-year-old female patient. This particular variation is exceedingly rare as it involves a typical SCA originating from the BA and a duplicate SCA emanating from the ICA, the latter of which has been reported only once in the extant literature. Understanding common and rare vascular structures closely associated with significant neurovascular elements is advantageous for clinicians when assessing atypical neuralgias or before conducting cerebral surgeries.

## Data Availability

No datasets were generated or analysed during the current study.
